# Correction: Unique characteristics of new complete blood count parameters, the Immature Platelet Fraction and the Immature Platelet Fraction Count, in dengue patients

**DOI:** 10.1371/journal.pone.0303463

**Published:** 2024-05-02

**Authors:** Ikkoh Yasuda, Nobuo Saito, Motoi Suzuki, Dorcas Valencia Umipig, Rontgene M. Solante, Ferdinand De Guzman, Ana Ria Sayo, Michio Yasunami, Nobuo Koizumi, Emi Kitashoji, Kentaro Sakashita, Chris Fook Sheng Ng, Chris Smith, Koya Ariyoshi

S1 and S2 Appendix Files are omitted from the list of Supporting Information. They can be viewed below.

## Supporting information

S1 Appendix Diagnostic criteria of CABIs. (PDF)

S2 Appendix Laboratory procedure for diagnoses of CABIs. (PDF)
