# Supplementary figures and images for: Unique characteristics of new complete blood count parameters, the Immature Platelet Fraction and the Immature Platelet Fraction Count, in dengue patients
Source: PLoS One. 2021 Nov 1;16(11):e0258936. doi: 10.1371/journal.pone.0258936 (PMC8559939; doi:10.1371/journal.pone.0258936)

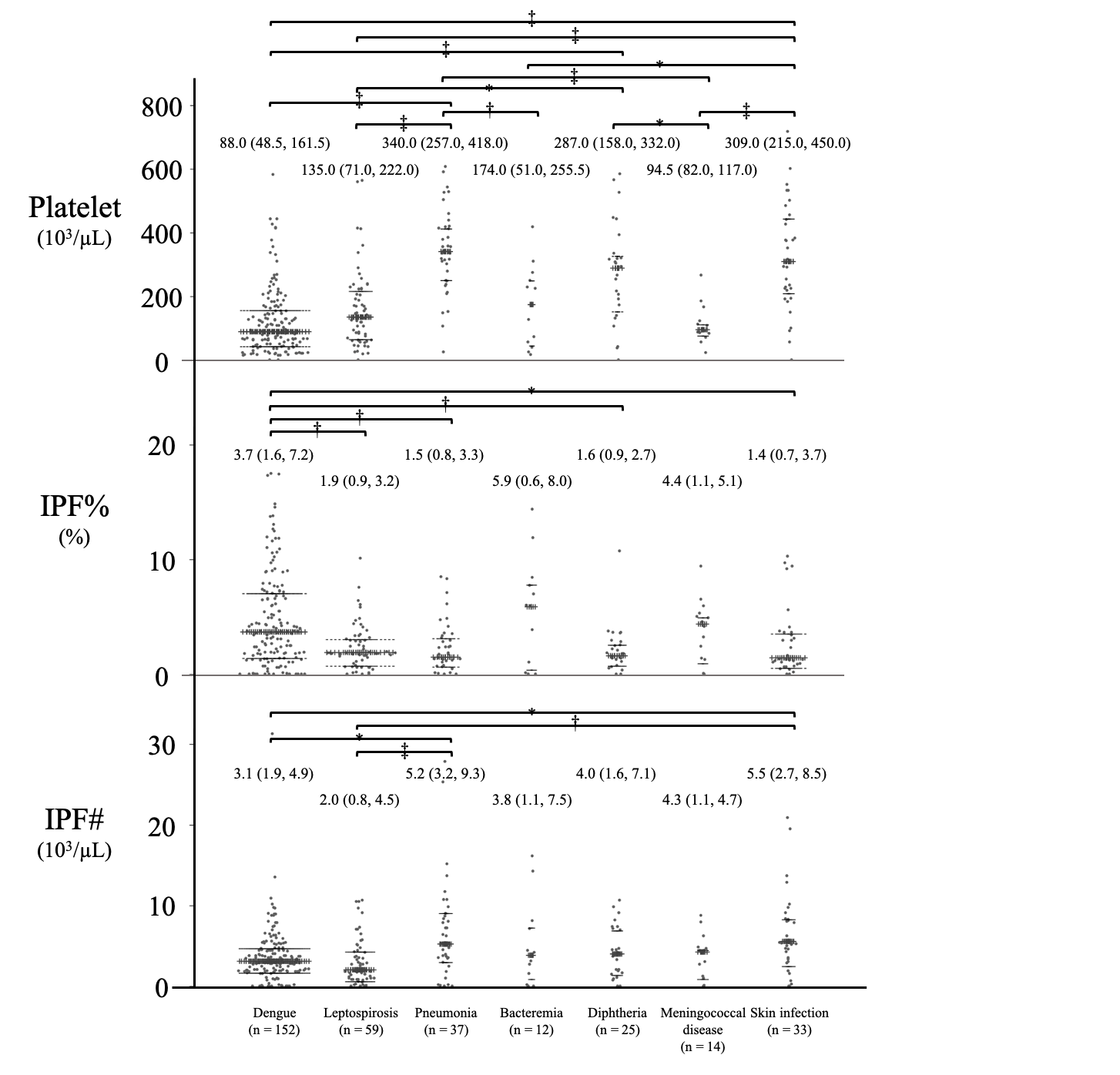

Supplement: S1 Fig — The lines show the median with interquartile ranges. Comparisons of each group were performed using the Kruskal-Wallis test followed by the Dunn’s post hoc test with Holm adjustment. *: P<0.05, †: P<0.01, ‡: P<0.001. IPF%: Immature Platelet Fraction, IPF#: Immature Platelet Fraction Count. (TIF) [file pone.0258936.s001.tif]

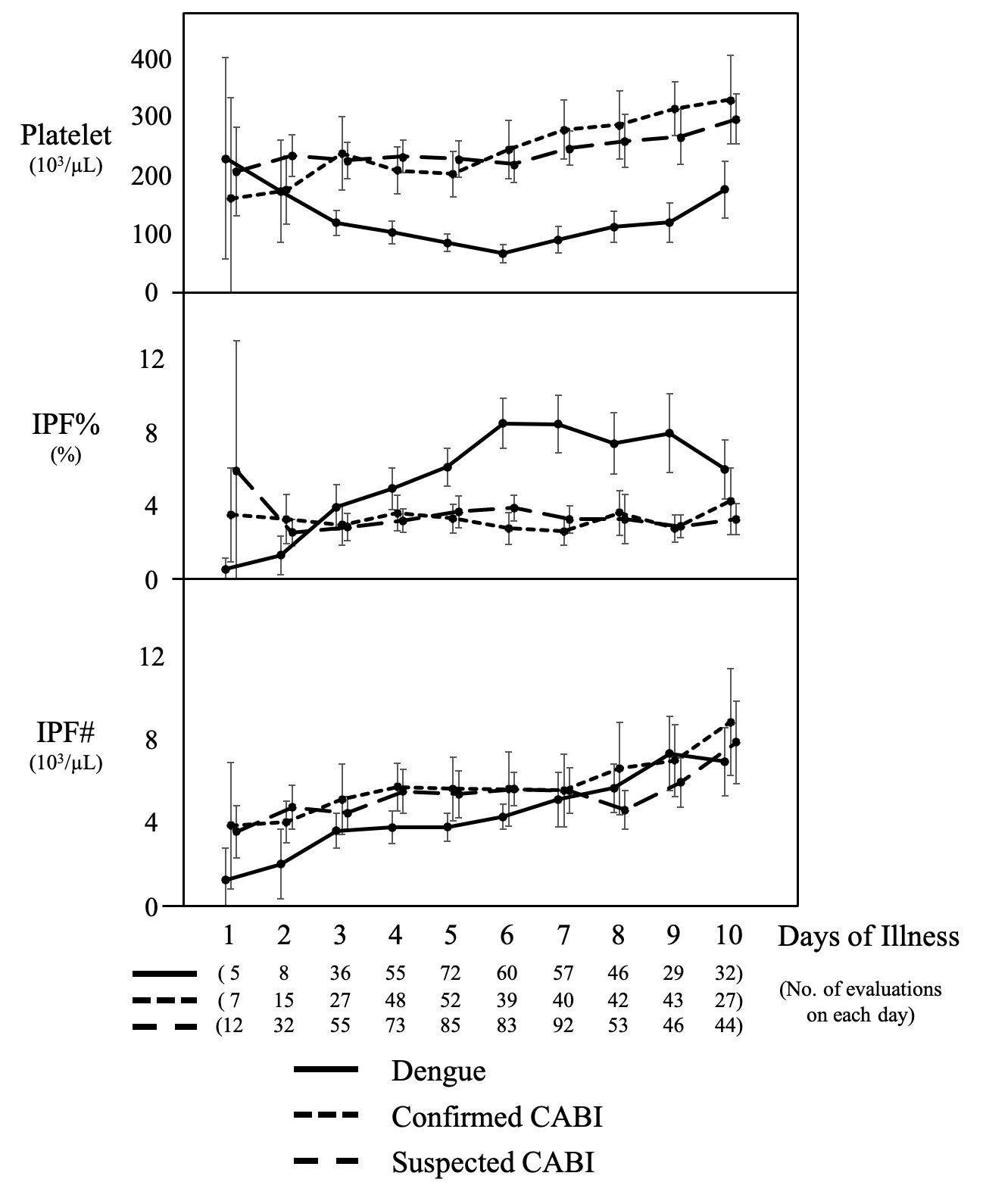

Supplement: S2 Fig — Data are expressed as mean with a 95% confidence interval shown by the error bars. The total number of evaluations included on each day is indicated on the X axis by each group. The mean of each platelet parameter by days of illness were calculated from the results of participants who underwent a blood test on each day. CABI: community acquired bacterial infection, IPF%: Immature Platelet Fraction, IPF#: Immature Platelet Fraction Count. (TIF) [file pone.0258936.s002.tif]

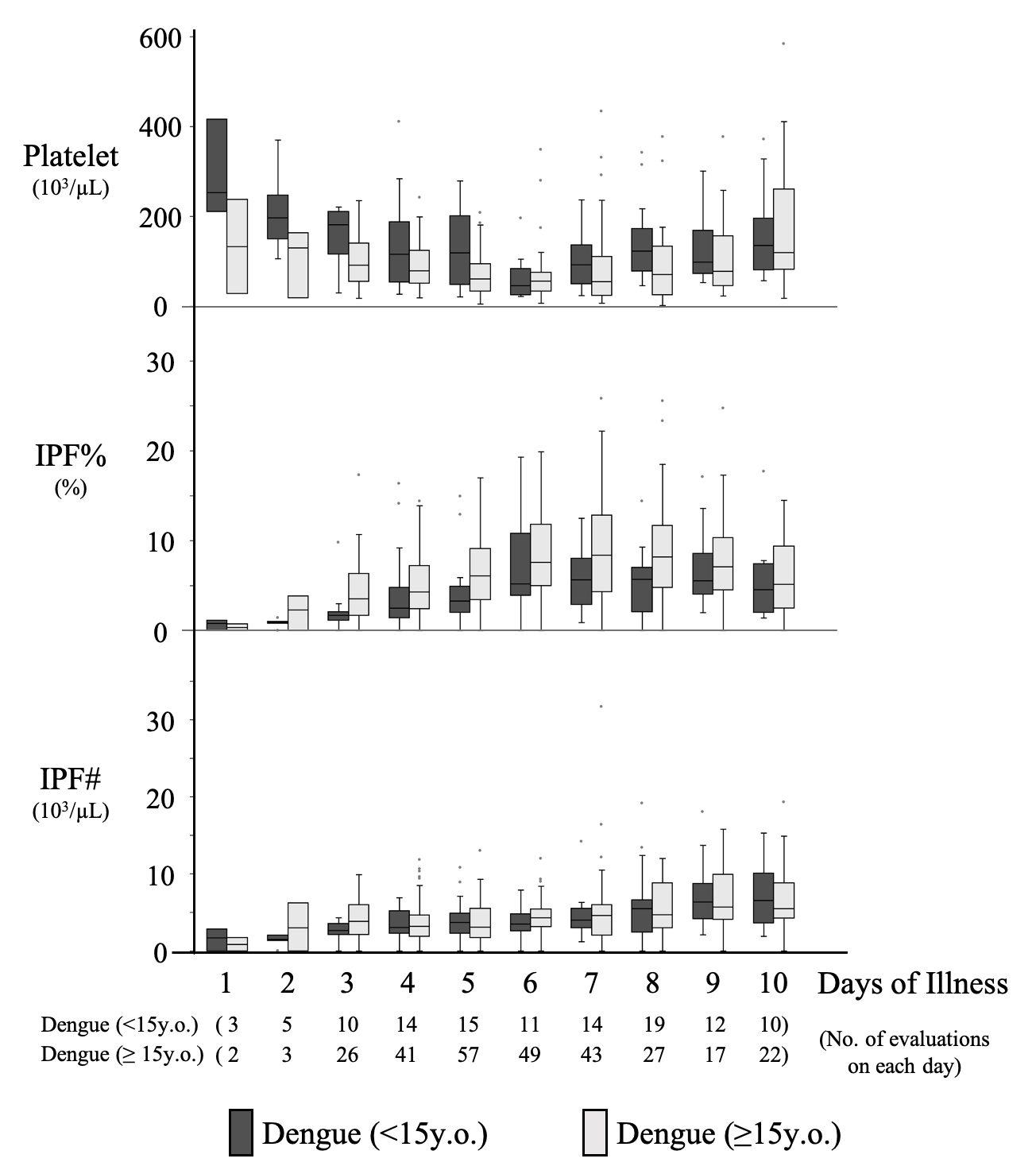

Supplement: S3 Fig — Box and whisker plots show the time course trends of platelet parameters in the dengue group by age from the first to tenth days of illness. Boxes show the median and interquartile values, whiskers represent upper and lower adjacent values and dots indicate outside values. The total number of evaluations included on each day is indicated on the X axis by each group. IPF%: Immature Platelet Fraction, IPF#: Immature Platelet Fraction Count. (TIF) [file pone.0258936.s003.tif]

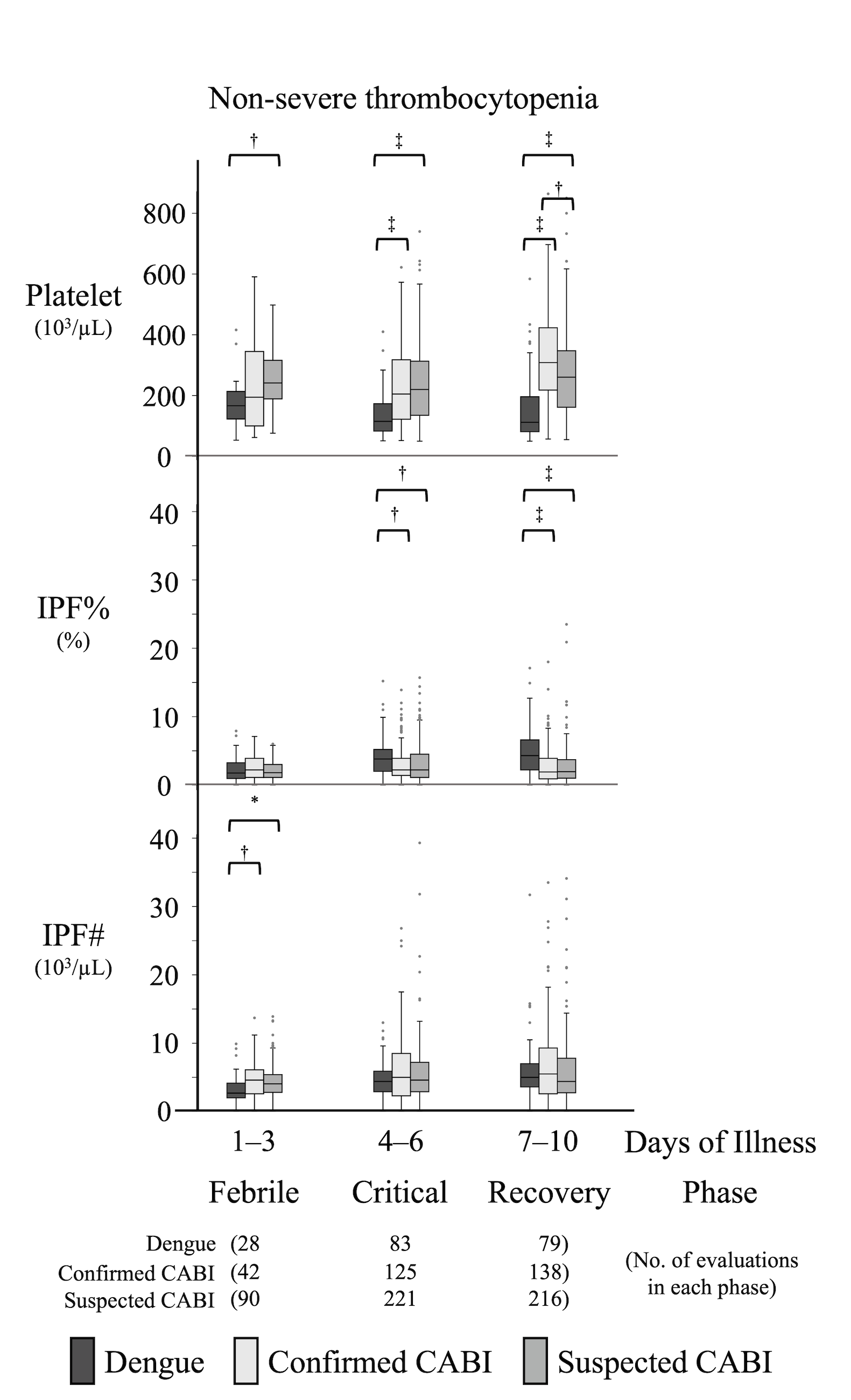

Supplement: S4 Fig — Box and whisker plots show the platelet parameters of dengue, confirmed CABI, and suspected CABI groups observed in specific time-phases: febrile phase (day 1 to 3), critical phase (day 4 to 6), and recovery phase (day 7 to 10). Non-severe thrombocytopenia was defined as a platelet nadir ≥50×103/μl during hospitalization. Boxes show the median and interquartile values, whiskers represent the upper and lower adjacent values and dots indicate outside values. Comparison of dengue, confirmed CABI, and suspected CABI groups were performed using the Kruskal-Wallis test followed by the Dunn’s post hoc test with Holm adjustment. *: P<0.05, †: P<0.01, ‡: P<0.001. CABI: community acquired bacterial infection, IPF%: Immature Platelet Fraction, IPF#: Immature Platelet Fraction Count. (TIF) [file pone.0258936.s004.tif]

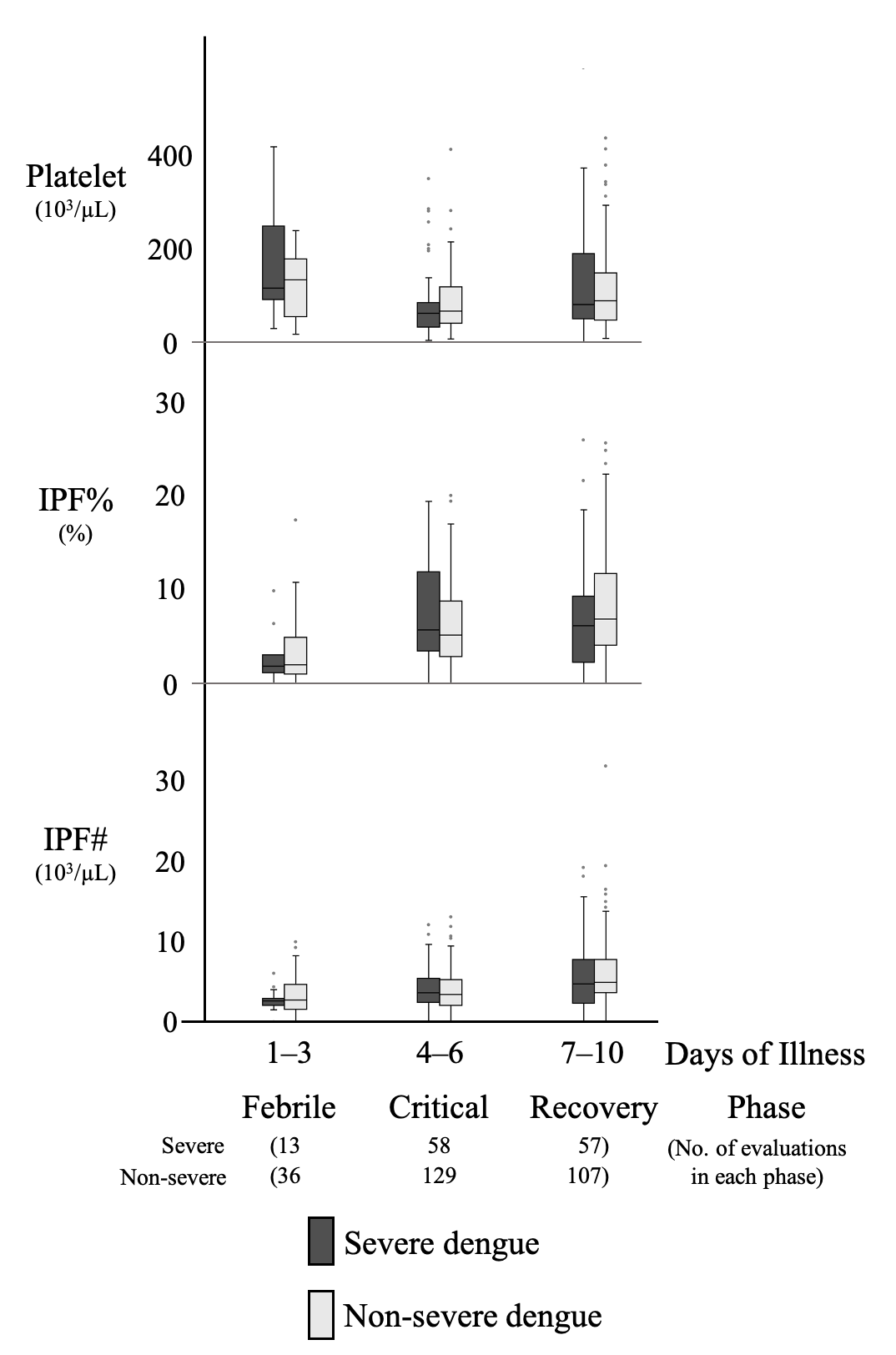

Supplement: S5 Fig — Box and whisker plots show the platelet parameters of severe and non-severe dengue groups observed in specific time-phases; febrile phase (day 1 to 3), critical phase (day 4 to 6) and recovery phase (day 7 to 10). Severe dengue was clinically defined in the “Disease definition” section. Boxes show the median and interquartile values, whiskers represent the upper and lower adjacent values and dots indicate outside values. Comparisons between each pair of dengue subgroups were performed using the Mann Whitney test. IPF%: Immature Platelet Fraction, IPF#: Immature Platelet Fraction Count. (TIF) [file pone.0258936.s005.tif]
