# Supplementary material for: Unique characteristics of new complete blood count parameters, the Immature Platelet Fraction and the Immature Platelet Fraction Count, in dengue patients
Source: PLoS One. 2021 Nov 1;16(11):e0258936. doi: 10.1371/journal.pone.0258936 (PMC8559939; doi:10.1371/journal.pone.0258936)
Supplement: S1 Table — A total number of 50 negative samples and 43 positive samples for flaB-nested PCR were tested in duplicate by rrs real-time PCR. The 50 negative samples for flaB-nested PCR were also negative for the microscopic agglutination test using paired sera. This rrs real-time PCR did not detect bacterial species other than Leptospira spp. described in published study [17]. rrs: 16S ribosomal RNA gene. (DOCX) [file pone.0258936.s006.docx]

|  |  | Real-time PCR (rrs probe) | |
| --- | --- | --- | --- |
|  |  | Negative | Positive |
| *flaB*-nested PCR | Negative | 99 | 1 |
|  | Positive | 0 | 86 |
