# Supplementary material for: Unique characteristics of new complete blood count parameters, the Immature Platelet Fraction and the Immature Platelet Fraction Count, in dengue patients
Source: PLoS One. 2021 Nov 1;16(11):e0258936. doi: 10.1371/journal.pone.0258936 (PMC8559939; doi:10.1371/journal.pone.0258936)
Supplement: Attachment — Submitted filename: PONE-D-21-17692 04_07_21 SL.pdf [file pone.0258936.s007.pdf]

PLOS ONE

Unique characteristics of new CBC parameters, the Immature Platelet Fraction and the Immature Platelet Fraction Count, in dengue patients.

--Manuscript Draft--

**Manuscript Number:**	PONE-D-21-17692
**Article Type:**	Research Article
**Full Title:**	Unique characteristics of new CBC parameters, the Immature Platelet Fraction and the Immature Platelet Fraction Count, in dengue patients.
**Short Title:**	Immature platelet evaluation in dengue.
**Corresponding Author:**	Ikkoh Yasuda<br>Fukushima Medical University<br>Fukushima City, JAPAN
**Keywords:**	Dengue; Community acquired bacterial infection; Immature Platelet Fraction; IPF; Immature Platelet Fraction Count; the Philippines
**Abstract:**	The advanced platelet parameters Immature Platelet Fraction and Immature Platelet Fraction Count have been implemented in clinical practice as measures of thrombopoietic activity, mainly in hematologic disorders that cause thrombocytopenia. Our purpose was to quantify platelet aspects of the thrombopoietic activity of dengue. ~~An observational study was conducted in an~~ infectious disease referral hospital in Metro Manila, the Philippines. We enrolled hospitalized patients at admission who were diagnosed with ~~an acute phase of~~ dengue or community acquired bacterial infection (CABI). Immature Platelet Fraction (IPF) and Immature Platelet Fraction Count were evaluated at admission and during hospitalization. A total of 606 patients were enrolled from May 1, 2017 to June 1, 2018. The participants consisted of 152 dengue, 180 confirmed CABI, and 274 suspected CABI patients. At admission, the percent IPF (IPF%) of the dengue was significantly higher than that of the confirmed CABI (median 3.7% versus 1.9%; p <0.001). In a time course evaluation, there was no significant difference of IPF% between dengue and confirmed CABI in the febrile phase (median 1.9% versus 2.4%; p = 0.488), however, the IPF% of dengue increased to be significantly higher than that of the confirmed CABI in the critical phase (median 5.2% versus 2.2%; p <0.001). Our study elucidated the unique characteristics and time-course trends of IPF percent and number (IPF#) in dengue. IPF% and IPF# are potentially valuable parameters in dengue and further investigation is required for the optimal usage in clinical practice.
**Order of Authors:**	Ikkoh Yasuda
	Nobuo Saito
	Motoi Suzuki
	Dorcas Valencia Umipig
	Rontgene M. Solante
	Ferdinand De Guzman
	Ana Ria Sayo
	Michio Yasunami
	Nobuo Koizumi
	Emi Kitashoji
	Kentaro Sakashita
	Chris Fook Sheng Ng
	Chris Smith
	Koya Ariyoshi

Powered by Editorial Manager® and ProduXion Manager® from Aries Systems Corporation

Additional Information:	
**Question**	**Response**
**Financial Disclosure**<br><br>Enter a financial disclosure statement that describes the sources of funding for the work included in this submission. Review the submission guidelines for detailed requirements. View published research articles from *PLOS ONE* for specific examples.<br><br>This statement is required for submission and **will appear in the published article** if the submission is accepted. Please make sure it is accurate.<br><br>**Unfunded studies**<br>Enter: *The author(s) received no specific funding for this work.*<br><br>**Funded studies**<br>Enter a statement with the following details:<br>• Initials of the authors who received each award<br>• Grant numbers awarded to each author<br>• The full name of each funder<br>• URL of each funder website<br>• Did the sponsors or funders play any role in the study design, data collection and analysis, decision to publish, or preparation of the manuscript?<br>• **NO** - Include this sentence at the end of your statement: *The funders had no role in study design, data collection and analysis, decision to publish, or preparation of the manuscript.*<br>• **YES** - Specify the role(s) played.<br><br>* typeset	This work was mostly supported by the Ministry of Education, Culture, Sports, Science, and Technology (MEXT), Government of Japan. Department of Clinical Medicine, Institute of Tropical Medicine, Nagasaki University has received research fund from Sysmex Corporation for the study: “An observational study of community acquired-bacteremia in San Lazaro Hospital, Manila, the Philippines” and the costs of testing for this study were partially covered by the research fund. The funders had no role in study design, data collection and analysis, decision to publish, or preparation of the manuscript.<br><br>URL of each funder website:<br>https://www.mext.go.jp/en/<br>https://www.sysmex.co.jp/en/index.html
**Competing Interests**<br><br>Use the instructions below to enter a competing interest statement for this submission. On behalf of all authors, disclose any competing interests that could be perceived to bias this work—acknowledging all financial support and any other relevant financial or non-financial competing interests.	I have read the journal's policy and the authors of this manuscript have the following competing interests: School of Tropical Medicine and Global Health, Nagasaki University was supplied with an automated hematology analyzer, Sysmex XN-1000, and the related reagents from Sysmex Corporation for the performance of this study. Each author has no competing interests individually.

This statement is required for submission and **will appear in the published article** if the submission is accepted. Please make sure it is accurate and that any funding sources listed in your Funding Information later in the submission form are also declared in your Financial Disclosure statement.<br><br>View published research articles from *PLOS ONE* for specific examples.<br><br>**NO authors have competing interests**<br><br>Enter: *The authors have declared that no competing interests exist.*<br><br>**Authors with competing interests**<br><br>Enter competing interest details beginning with this statement:<br><br>*I have read the journal's policy and the authors of this manuscript have the following competing interests: [insert competing interests here]*<br><br>* typeset	
**Ethics Statement**<br><br>Enter an ethics statement for this submission. This statement is required if the study involved:<br><br>• Human participants<br>• Human specimens or tissue<br>• Vertebrate animals or cephalopods<br>• Vertebrate embryos or tissues<br>• Field research<br><br>Write "N/A" if the submission does not require an ethics statement.<br><br>General guidance is provided below. Consult the submission guidelines for detailed instructions. **Make sure that all information entered here is included in the**	This work was conducted as a sub-study of a main study: "An observational study of community acquired bacteremia in San Lazaro Hospital, Manila, The Philippines". Ethical approval for the main study was obtained from the Research Ethical and Review Unit of San Lazaro Hospital, the Philippines (number: SLH-RERU-2015-005-E) and the Institutional Review Board of the Institute of Tropical Medicine, Nagasaki University, Japan (number: 150226136-4). Written informed consent was obtained from guardians or caregivers for patients aged under 18 years of age, illiterate or unconscious at presentation. For all others, written consent was obtained from the participants.

**Methods section of the manuscript.**<br><br>**Format for specific study types**<br><br>**Human Subject Research (involving human participants and/or tissue)**<br>• Give the name of the institutional review board or ethics committee that approved the study<br>• Include the approval number and/or a statement indicating approval of this research<br>• Indicate the form of consent obtained (written/oral) or the reason that consent was not obtained (e.g. the data were analyzed anonymously)<br><br>**Animal Research (involving vertebrate animals, embryos or tissues)**<br>• Provide the name of the Institutional Animal Care and Use Committee (IACUC) or other relevant ethics board that reviewed the study protocol, and indicate whether they approved this research or granted a formal waiver of ethical approval<br>• Include an approval number if one was obtained<br>• If the study involved *non-human primates*, add *additional details* about animal welfare and steps taken to ameliorate suffering<br>• If anesthesia, euthanasia, or any kind of animal sacrifice is part of the study, include briefly which substances and/or methods were applied<br><br>**Field Research**<br><br>Include the following details if this study involves the collection of plant, animal, or other materials from a natural setting:<br>• Field permit number<br>• Name of the institution or relevant body that granted permission	
**Data Availability**<br><br>Authors are required to make all data underlying the findings described fully available, without restriction, and from the time of publication. PLOS allows rare exceptions to address legal and ethical concerns. See the PLOS Data Policy and FAQ for detailed information.	Yes - all data are fully available without restriction

Powered by Editorial Manager® and ProduXion Manager® from Aries Systems Corporation

A Data Availability Statement describing where the data can be found is required at submission. Your answers to this question constitute the Data Availability Statement and **will be published in the article**, if accepted.<br><br>**Important:** Stating 'data available on request from the author' is not sufficient. If your data are only available upon request, select 'No' for the first question and explain your exceptional situation in the text box.<br><br>Do the authors confirm that all data underlying the findings described in their manuscript are fully available without restriction?	
**Describe where the data may be found in full sentences. If you are copying our sample text, replace any instances of XXX with the appropriate details.**<br><br>• If the data are **held or will be held in a public repository**, include URLs, accession numbers or DOIs. If this information will only be available after acceptance, indicate this by ticking the box below. For example: *All XXX files are available from the XXX database (accession number(s) XXX, XXX.*).<br>• If the data are all contained **within the manuscript and/or Supporting Information files**, enter the following: *All relevant data are within the manuscript and its Supporting Information files.*<br>• If neither of these applies but you are able to provide **details of access elsewhere**, with or without limitations, please do so. For example:<br><br>*Data cannot be shared publicly because of [XXX]. Data are available from the XXX Institutional Data Access / Ethics Committee (contact via XXX) for researchers who meet the criteria for access to confidential data.*<br><br>*The data underlying the results*	All relevant data are within the manuscript and its Supporting Information files.

presented in the study are available from (include the name of the third party and contact information or URL).<br>• This text is appropriate if the data are owned by a third party and authors do not have permission to share the data.<br><br>* typeset	
Additional data availability information:	

Powered by Editorial Manager® and ProduXion Manager® from Aries Systems Corporation

Manuscript
Click here to access/download;Manuscript;IPF_PLOSONE_manuscript.docx

Full title: Unique characteristics of new CBC parameters, the Immature Platelet Fraction and the Immature Platelet Fraction Count, in dengue patients.

Short title: Immature platelet evaluation in dengue.

Ikkoh Yasuda[1,2,3], Nobuo Saito[1,2,4*], Motoi Suzuki[2,5], Dorcas Valencia Umipig[6], Rontgene M. Solante[6], Ferdinand De Guzman[6], Ana Ria Sayo[6], Michio Yasunami[2,7], Nobuo Koizumi[8], Emi Kitashoji[2], Kentaro Sakashita[1,9], Chris Fook Sheng Ng[1], Chris Smith[1,10], Koya Ariyoshi[1,2]

[1]School of Tropical Medicine and Global Health, Nagasaki University, Nagasaki, Japan

[2]Department of Clinical Medicine, Institute of Tropical Medicine, Nagasaki University, Nagasaki, Japan

[3]Department of General Internal Medicine and Clinical Infectious Diseases, Fukushima Medical University, Fukushima, Japan

[4]Department of Microbiology, Oita University Faculty of Medicine, Oita, Japan

[5]Infectious Disease Surveillance Center, National Institute of Infectious Diseases, Tokyo, Japan

[6]San Lazaro Hospital, Manila, The Philippines

[7]Saga-Ken Medical Centre Koseikan, Saga, Japan

[8]Department of Bacteriology I, National Institute of Infectious Diseases, Toyama, Shinjuku-ku, Tokyo, Japan

[9]Department of Basic Mycobacteriology, Graduate School of Biomedical Science, Nagasaki University, Nagasaki, Japan

[10]Department of Clinical Research, London School of Hygiene and Tropical Medicine, London, United Kingdom

*Corresponding author

E-mail: nobuosaito@oita-u.ac.jp (NS)

Author contributions

Conceptualization: Nobuo Saito, Motoi Suzuki, Koya Ariyoshi

Data curation: Nobuo Saito, Ikkoh Yasuda

Formal analysis: Nobuo Saito, Ikkoh Yasuda, Chris Fook Sheng Ng

Funding acquisition: Nobuo Saito, Koya Ariyoshi

Investigation: Nobuo Saito, Ikkoh Yasuda, Dorcas Valencia Umipig, Rontgene M. Solante, Ferdinand De Guzman, Ana Ria Sayo, Kentaro Sakashita

Methodology: Nobuo Saito, Motoi Suzuki, Michio Yasunami, Nobuo Koizumi, Emi Kitashoji, Koya Ariyoshi

Project Administration: Nobuo Saito

Resources: Nobuo Saito, Ikkoh Yasuda, Dorcas Valencia Umipig, Rontgene M. Solante, Ferdinand De Guzman, Ana Ria Sayo, Michio Yasunami, Nobuo Koizumi, Emi Kitashoji, Kentaro Sakashita

Software: Ikkoh Yasuda, Nobuo Saito

Supervision: Nobuo Saito, Motoi Suzuki, Koya Ariyoshi

Validation: Nobuo Saito, Ikkoh Yasuda

Visualization: Ikkoh Yasuda, Nobuo Saito

Writing – Original Draft Preparation: Ikkoh Yasuda

Writing – Review & Editing: Nobuo Saito, Chris Fook Sheng Ng, Chris Smith, Koya Ariyoshi

Abstract

The advanced platelet parameters Immature Platelet Fraction and Immature Platelet Fraction Count have been implemented in clinical practice as measures of thrombopoietic activity, mainly in hematologic disorders that cause thrombocytopenia. Our purpose was to quantify platelet aspects of the thrombopoietic activity of dengue. An observational study was conducted in an infectious disease referral hospital in Metro Manila, the Philippines. We enrolled hospitalized patients at admission who were diagnosed with an acute phase of dengue or community acquired bacterial infection (CABI). Immature Platelet Fraction (IPF) and Immature Platelet Fraction Count were evaluated at admission and during hospitalization. A total of 606 patients were enrolled from May 1, 2017 to June 1, 2018. The participants consisted of 152 dengue, 180 confirmed CABI, and 274 suspected CABI patients. At admission, the percent IPF (IPF%) of the dengue was significantly higher than that of the confirmed CABI (median 3.7% versus 1.9%; $p < 0.001$). In a time course evaluation, there was no significant difference of IPF% between dengue and confirmed CABI in the febrile phase (median 1.9% versus 2.4%; $p = 0.488$), however, the IPF% of dengue increased to be

significantly higher than that of the confirmed CABI in the critical phase (median 5.2% versus 2.2%; p <0.001). Our study elucidated the unique characteristics and time-course trends of IPF percent and number (IPF#) in dengue. IPF% and IPF# are potentially valuable parameters in dengue and further investigation is required for the optimal usage in clinical practice.

Introduction

Thrombocytopenia induced by dengue is typical around the time of defervescence and is recognized as a potential indicator of clinical worsening according to the 2009 WHO guidelines. [1,2] However, the underlying pathophysiological mechanisms involved in dengue-induced thrombocytopenia remain controversial. [3] Recently, the Immature Platelet Fraction (IPF%) and Immature Platelet Fraction Count (IPF#) have been recognized as measures of thrombopoietic activity. [4,5] Conceptually, IPF% and IPF# correspond respectively to the percentage and absolute number of immature platelets in peripheral blood. ~~Using these platelet parameters, studies have investigated thrombocytopenia of hematologic disorders and conditions such as immune thrombocytopenia (ITP), thrombotic thrombocytopenic purpura (TTP), aplastic anemia, and chemotherapy-induced thrombocytopenia.~~ [4,6] It is assumed that an increased IPF% indicates a consumptive or destructive thrombocytopenic status; whereas a normal or decreased IPF% suggests decreased platelet production in bone marrow. [7-9] IPF# is considered to reflect real-time platelet production. [5] Although IPF% and IFP# have been implemented in wider clinical settings, the benefit of evaluating these

parameters in dengue has not been fully determined. Therefore, this study aimed to investigate the thrombopoietic activity of dengue by quantifying IPF% and IPF# and to elucidate their characteristics by comparing with those parameters of community acquired bacterial infection (CABI).

Materials and Methods

This analysis was implemented under an existing study: “An observational study of community acquired-bacteremia in San Lazaro Hospital, Manila, The Philippines”. San Lazaro Hospital is a national tertiary referral and leading training hospital for infectious diseases and has a 500-bed capacity. This substudy comprised patients who were enrolled in the main study from May 1, 2017 to June 1, 2018 and satisfied eligibility criteria as follows: (i) admitted to San Lazaro Hospital, (ii) aged ≥ 1 year at admission, and (iii) having acute onset of fever (≤ 21 days) at admission. The participants were subsequently categorized into the three groups - confirmed dengue, confirmed CABI, or suspected CABI – following the disease definitions described below. Because the

confirmed CABI group was potentially weighted toward bacterial infections diagnosable at the study site, the suspected CABI was included as a category. Subjects were excluded if suspected of active tuberculosis or diagnosed with human immunodeficiency virus (HIV) infection. Participants were also excluded if unable to provide blood for a complete blood count (CBC) test at admission or who received blood transfusion during hospitalization.

Disease definition

The confirmed diagnoses of each disease were defined as follows: dengue diagnosis was when the nonstructural protein 1 (NS1) antigen was positive and/or dengue reverse transcriptase polymerase chain reaction (PCR) was positive without a positive blood culture result. Patients were also diagnosed with dengue if positive for dengue IgM without other suspected diagnoses or positive blood culture results. Bacteremia was diagnosed by positive blood culture. Diphtheria was diagnosed when diphtheria culture and/or tox-gene PCR from nasopharyngeal or oropharyngeal sample were positive.

Meningococcal disease was diagnosed when meningococcus culture and/or PCR from blood or skin lesion samples were positive. Leptospirosis was diagnosed when one of the following were positive: culture with Korthof's medium, Patoc-IgM enzyme linked immunosorbent assay (ELISA) (positivity was defined as described [10]), or real-time PCR assay (see the details described later in this section). X-ray confirmed pneumonia was diagnosed when patients had acute onset of respiratory symptoms and radiographic shadowing in lung fields without other suspected infection focus or positive blood culture result. Skin infection was diagnosed when patients had acute onset of symptoms compatible with skin infection without other suspected infection focus or positive blood culture result. Suspected CABI was clinically diagnosed after excluding dengue infection but unable to reach a confirmed CABI diagnosis as described above.

Severe thrombocytopenia was defined as platelets <50×10^3/µl at admission or a platelet nadir <50×10^3/µl during hospitalization in a time course evaluation.

Severe dengue was clinically defined as having at least one of the following conditions at admission: systolic blood pressure ≤90 mmHg, desaturation requiring oxygenation,

aspartate aminotransferase ≥ 1000 IU/L and/or alanine transaminase ≥1000 IU/L, or impaired consciousness.

NS1 antigen and dengue IgM were tested using a SD BIOLINE Dengue Duo™ kit (Standard Diagnostics, Korea). The blood culture results were detected using a BacT/ALERT automated system (BioMerieu[illegible]nd isolates were identified by Matrix Assisted Laser Desorption/Ionization Time of Flight Mass Spectrometry (MALDI-TOF MS) or standard microbiological methods. ~~Primer pairs and probes that target leptospiral 16S ribosomal RNA gene (*rrs*) were newly designed for a real-time PCR assay (F3C, 5’-TCATTGGGCGTAAAGGGTG-3’; B3C, 5’-TCAGTTTTAGGCCAGCAAGTC-3’; Probe, 56-FAM/AGAGGCAAG/ZEN/TGGAATTCCAGGTG/BHQ), and the performance compared with *flaB* nested PCR as shown in S1 Table. The details of other PCR and ELISA method used in this study were as published. [10-14]~~

Laboratory procedure for IPF% and IPF#

Peripheral blood samples were drawn into tubes containing ethylene diamine tetra-acetic acid (EDTA). In addition to regular CBC parameters, IPF% and IPF# were evaluated using an automated hematology analyzer (Sysmex XN-1000, Sysmex, Kobe, Japan). The analyzer detects immature platelets, which are larger in size and contain more RNA than mature platelets, by staining intracellular RNA with oxazine fluorescent dye. IPF% is expressed as a percentage representing the ratio of the absolute number of immature platelets to the total number of platelets. IPF# represents the absolute number of immature platelets per unit volume.

Data collection and statistical analysis

~~Clinical information consisting of~~ age, demographic data and past medical history were documented at admission ~~by medical doctors and research nurses using a structured report form~~. ~~The first blood collection was conducted on admission day and the following blood collections were done simultaneously with regular CBC check as frequently as required for clinical management.~~ ~~All clinical information and blood analysis data were recorded into an electronic database and analyzed.~~ To evaluate the

time course trend of platelet parameters, the representative values of each parameter by day of illness were calculated using ~~only~~ the results of participants who underwent blood tests on ~~the~~ day of illness ~~because~~ the timing of blood collection varied ~~by~~ participants and the sequential day-by-day test results were not available for everyone. We defined the days of illness as the duration from the day of onset defined as day 0 of illness to the day of interest. The clinicians were not blinded to the laboratory results. Baseline characteristics were compared using Fisher’s exact test for categorical variables or the Mann Whitney test for continuous variables. Comparison of dengue, confirmed CABI, and suspected CABI groups were performed using the Kruskal-Wallis test followed by the Dunn’s post hoc test with Holm adjustment. Pairwise comparisons were performed using the Mann Whitney test. P values of less than 0.05 were considered statistically significant. All analyses were performed using Stata version 14.2 (Stata Corp., College Station, TX, USA).

Ethical Issues

This work was conducted as a sub-study of a main study: “An observational study of community acquired bacteremia in San Lazaro Hospital, Manila, The Philippines”. Ethical approval for the main study was obtained from the Research Ethical and Review Unit of San Lazaro Hospital, the Philippines (number: SLH-RERU-2015-005-E) and the Institutional Review Board of the Institute of Tropical Medicine, Nagasaki University, Japan (number: 150226136-4). Written informed consent was obtained from guardians or caregivers for patients aged under 18 years of age, illiterate or unconscious at presentation. For all others, written consent was obtained from the participants.

Results

1. Basic characteristics

A total of 606 patients were eligible after excluding 19 subjects who satisfied the inclusion criteria but received blood transfusion during treatment and one participant whose CBC test result was not available at admission. The participants consisted of 152 dengue, 180 confirmed CABI and 274 suspected CABI. The confirmed diagnosis in the

CABI patients included 59 leptospirosis, 37 X-ray confirmed pneumonia, 12 bacteremia, 25 diphtheria, 14 meningococcal disease, and 33 skin infection. The clinical diagnoses in the suspected CABI patients consisted of 72 pneumonia, 26 enteric fever, 23 urinary tract infection, 10 leptospirosis, 8 central nervous system infection, 4 abdominal infection, 1 meningococcal disease, 1 septic rash, and 129 undiagnosable infectious disease. The median ages were 19.0 years (interquartile range (IQR): 13.0, 25.0) in the dengue group, 20.5 years (IQR: 9.0, 33.5) in the confirmed CABI group and 19.0 years (IQR: 10.0, 32.0) in the suspected CABI group, and there were no significant differences between the dengue and each CABI group.

Table 1. Basic characteristics of participants at admission.

	Dengue (n=152)	Confirmed CABI (n=180)	p*	Suspected CABI (n=274)	p*
	n (%) or median (IQR)	n (%) or median (IQR)		n (%) or median (IQR)	
Age	19.0 (13.0, 25.0)	20.5 (9.0, 33.5)	0.628	19.0 (10.0, 32.0)	0.985
Male	100 (65.8)	133 (73.9)	0.118	175 (63.9)	0.751
Mortality	1 (0.7)	22 (12.2)	<0.001	9 (3.3)	0.104
~~Underlying diseases~~	17 (11.2)	42 (23.3)	0.004	78 (28.5)	<0.001
Days of illness at admission					
0–3	40 (26.3)	42 (23.3)	0.027	75 (27.4)	<0.001
4,5	71 (46.7)	63 (35.0)		78 (28.5)	

≥6		38 (25.0)	72 (40.0)		114 (41.6)	
unknown onset		3 (2.0)	3 (1.7)		7 (2.6)	
**Routine hematology at admission**						
Platelets	(10^3/µL)	88.0 (48.5, 161.5)	225.5 (100.0, 342.5)	<0.001	221.0 (141.0, 316.0)	<0.001
IPF%	(%)	3.7 (1.6, 7.2)	1.9 (0.9, 3.5)	<0.001	1.9 (1.0, 3.7)	<0.001
IPF#	(10^3/µL)	3.1 (1.9, 4.9)	3.9 (1.5, 7.0)	0.084	3.6 (2.4, 5.8)	0.002
WBC	(10^3/µL)	4.2 (2.6, 6.5)	12.4 (8.3, 16.4)	<0.001	8.3 (5.7, 12.6)	<0.001
Neutrophils	(%)	55.2 (35.5, 70.8)	79.0 (67.0, 87.5)	<0.001	69.7 (54.6, 79.4)	<0.001
Lymphocytes	(%)	33.3 (20.0, 51.8)	12.5 (5.5, 21.5)	<0.001	20.2 (12.1, 32.8)	<0.001
Monocytes	(%)	9.4 (6.6, 13.2)	6.4 (4.5, 8.8)	<0.001	8.2 (5.9, 10.6)	0.002

Eosinophils	(%)	0.4 (0.0, 1.0)	0.3 (0.0, 1.5)	0.136	0.3 (0.0, 1.9)	0.085
RBC	(10^6/µL)	5.1 (4.7, 5.6)	4.5 (4.1, 4.9)	<0.001	4.7 (4.4, 5.2)	<0.001
Hemoglobin	(g/dL)	138.0 (127.0, 152.5)	122.0 (108.5, 132.0)	<0.001	126.0 (115.0, 140.0)	<0.001
Hematocrit	(%)	40.9 (38.7, 45.0)	36.9 (33.0, 40.0)	<0.001	38.5 (34.6, 42.2)	<0.001
AST	(IU/L)	97.0 (57.0, 168.0)	32.0 (22.0, 59.0)	<0.001	48.5 (28.0, 110.5)	<0.001
ALT	(IU/L)	58.0 (32.0, 118.0)	29.5 (20.0, 57.0)	<0.001	44.0 (22.0, 82.0)	0.029
BUN	(mg/dl)	9.8 (7.1, 12.6)	15.1 (10.2, 39.0)	<0.001	9.4 (6.9, 13.8)	0.823
Creatinine	(mg/dl)	0.8 (0.6, 0.9)	1.1 (0.6, 2.3)	<0.001	0.8 (0.6, 1.1)	0.366
CRP	(mg/L)	0.6 (0.1, 1.4)	11.5 (5.3, 16.0)	<0.001	2.8 (0.9, 9.4)	<0.001
PCT	(ng/mL)	0.6 (0.3, 1.2)	2.2 (0.3, 10.0)	<0.001	0.4 (0.1, 1.8)	0.052

* Between dengue and each CABI group using Fisher's exact test for categorical variables or Mann Whitney test for continuous variables.

Missing number of participants in the dengue, confirmed CABI and suspected CABI group for –AST = 89, 107, 170; ALT = 78, 84, 156; BUN = 83, 81, 153; –Cre = 70, 71, 151; PCT = 6, 6, 17. CABI: community acquired bacterial infection, IQR: interquartile range, IPF%: Immature Platelet Fraction, IPF#: Immature Platelet Fraction Count.

2. Comparison of platelet, IPF%, and IPF# at admission among the dengue, confirmed CABI, and suspected CABI groups

Fig 1 shows the comparison of platelet, IPF% and IPF# at admission among the dengue, confirmed CABI and suspected CABI groups. The median day of illness at admission was 5 in all groups and there was no significant difference among groups. (A) shows a comparison including all participants. Platelet counts of the dengue group were significantly lower than those of the confirmed CABI (median $88.0\times10^3/\mu L$ *vs.* $225.5\times10^3/\mu L$; $p < 0.001$) and suspected CABI groups (median $88.0\times10^3/\mu L$ *vs.* $221.0\times10^3/\mu L$; $p < 0.001$). The IPF% of the dengue group was significantly higher than those of the confirmed CABI (median 3.7% versus 1.9%; $p < 0.001$) and suspected CABI groups (median 3.7% *vs.* 1.9%; $p < 0.001$). Although there was no significant difference of IPF# between the dengue group and the confirmed CABI group (median $3.1\times10^3/\mu L$ versus $3.9\times10^3/\mu L$; $p = 0.057$), the IPF# of the dengue group was significantly lower than that of the suspected CABI group (median $3.1\times10^3/\mu L$ versus $3.6\times10^3/\mu L$; $p = 0.005$). The platelet parameters of each confirmed CABI are shown

separately in S1 Fig. (B) shows a comparison including only the subgroups with severe thrombocytopenia $<50\times10^3$/μL at admission. The median day of illness at admission was 5 for the three severe thrombocytopenia groups and there was no significant difference among groups. There was no significant difference of platelet counts among the three groups. Although the IPF% of the dengue group remained significantly higher than that of the confirmed CABI group (median 9.5% versus 3.4%; $p = 0.005$), there was no significant difference of IPF% between the dengue and the suspected CABI group (median 9.5% versus 6.9%; $p = 0.247$). The IPF# of the dengue group was significantly higher than that of the confirmed CABI group (median 2.5×10^3/μL versus 0.8×10^3/μL; $P = 0.007$) but there was no significant difference of the IPF# between the dengue and the suspected CABI group (median 2.5×10^3/μL vs. 1.6×10^3/μL; $p=0.123$).

Fig 1. Comparison of platelet, IPF% and IPF# among dengue, confirmed CABI, and suspected CABI groups at admission. Horizontal lines show the median and interquartile ranges. (A) shows a comparison including all participants and (B) shows a comparison including only the subgroups with severe thrombocytopenia $<50\times10^3$/μL at

admission. Comparison of dengue, confirmed CABI and suspected CABI groups were performed using Kruskal-Wallis test followed by the Dunn's post hoc test with Holm adjustment. *: P<0.05, †: P<0.01, ‡: P<0.001. CABI: community acquired bacterial infection, IPF%: Immature Platelet Fraction, IPF#: Immature Platelet Fraction Count.

3. Time course of platelet parameters by days of illness

Fig 2 shows the time course trends of platelet, IPF% and IPF# in the dengue, confirmed CABI and suspected CABI groups from the first to tenth days of illness. The platelet counts of the dengue group decreased from day 1, with a minimum observed on the sixth day of illness before recovering to a normal level approximately on the tenth day of illness. The platelet count of the two CABI groups shared a similar pattern, with a slightly increasing trend for the whole duration. The IPF% of the dengue group increased from the start and peaked on the sixth and seventh days, mirroring the pattern of the platelet count. The IPF% of the two CABI groups remained level during the whole period. The IPF# of all three groups showed a gradual increasing trend although the dengue group was slightly steeper. The line plot of the same data is shown in S2

Fig. The time courses of the platelet parameters in the dengue group are shown by age groups in S3 Fig and their trends were similar.

Fig 2. Time course trends of platelet parameters in each group from the first to tenth days of illness. Box and whisker plots show the time course trends of platelet parameters in each group from the first to tenth days of illness. Boxes show the median and interquartile values, whiskers represent the upper and lower adjacent values and dots indicate outside values. The total numbers of evaluations included on each day are indicated on the X axis by each group. CABI: community acquired bacterial infection, IPF%: Immature Platelet Fraction, IPF#: Immature Platelet Fraction Count.

4. Comparison of platelet parameters among dengue, confirmed CABI, and suspected CABI groups by specific time-phases

Fig 3 shows the comparison of platelet parameters among dengue, confirmed CABI, and suspected CABI groups by three time-phases; febrile phase (day 1 to 3), critical

phase (day 4 to 6), and recovery phase (day 7 to 10). (A) shows a comparison including all participants. The platelet counts of the dengue group were significantly lower than those of both the confirmed and suspected CABI groups in all phases. There was no significant difference of IPF% between dengue group and confirmed CABI group in the febrile phase (median 1.9% versus 2.4%; p = 0.488), however, the IPF% of dengue group increased to be significantly higher than that of the confirmed CABI in the critical phase (median 5.2% versus 2.2%; p <0.001) and recovery phase (median 6.5% versus 2.2%; p <0.001). The IPF% of the dengue group was also significantly higher than that of the suspected CABI groups in the critical and recovery phases. The IPF# of the dengue group was significantly lower than those of the confirmed and suspected CABI groups in the febrile and critical phases. (B) shows a comparison including only the subgroups with severe thrombocytopenia (defined as platelet nadir $<50\times10^3/\mu l$) during hospitalization. There were 67 dengue, 21 confirmed CABI and 25 suspected CABI subjects with severe thrombocytopenia. The IPF% of the dengue group remained significantly higher than that of the confirmed CABI group in the critical phase (median 8.2% versus 2.0%; p <0.001) and recovery phases (median 9.2% versus 5.3%; p = 0.02)

even though there was no significant difference of platelet counts between the groups in the same phases. The IPF% of the dengue group also remained significantly higher than that of the suspected CABI group in the recovery phases. The IPF# of the confirmed CABI was significantly lower than that of the dengue group in the critical phase despite no significant difference of platelet counts between the groups in that same phase.

Fig 3. Comparison of platelet parameters among dengue, confirmed CABI, and suspected CABI groups by specific time-phases. Box and whisker plots show the platelet parameters of dengue, confirmed CABI, and suspected CABI groups observed in specific time-phases: febrile phase (day 1 to 3), critical phase (day 4 to 6), and recovery phase (day 7 to 10). (A) shows a comparison including all participants and (B) shows a comparison including only the subgroups with severe thrombocytopenia defined as platelet nadir $<50\times10^3/\mu l$ during hospitalization. Boxes show the median and interquartile values, whiskers represent the upper and lower adjacent values and dots indicate outside values. Comparison of dengue, confirmed CABI, and suspected CABI groups were performed using the Kruskal-Wallis test followed by the Dunn’s post hoc

test with Holm adjustment. *: P<0.05, †: P<0.01, ‡: P<0.001. CABI: community acquired bacterial infection, IPF%: Immature Platelet Fraction, IPF#: Immature Platelet Fraction Count.

5. Comparison of platelet parameters between the severe thrombocytopenia dengue group and the non-severe thrombocytopenia dengue group by specific time-phases

Fig 4 shows the comparison of platelet parameters between the severe thrombocytopenia dengue group defined as platelet nadir $<50\times10^3$/μl during hospitalization and the non-severe thrombocytopenia dengue group by specific time-phases. The platelet counts of the severe thrombocytopenia dengue group were significantly lower than those of the non-severe thrombocytopenia dengue group in all phases. The IPF% of the severe thrombocytopenia dengue group was significantly higher than that of the non-severe thrombocytopenia dengue group in the critical phase (median 8.2% versus 3.8%; p <0.001) and recovery phases (median 9.2% versus 4.3%; p <0.001). The IPF# of the severe thrombocytopenia dengue group was significantly

lower than that of the non-severe thrombocytopenia dengue group only in the critical phases. S4 Fig shows a comparison between the severe and non-severe dengue groups (defined in the “Disease definition” section) There were 50 severe dengue subjects. For all platelet parameters there was no significant difference between the severe dengue and non-severe dengue groups in all phases.

Fig 4. Comparison of platelet parameters between the severe and non-severe dengue thrombocytopenia groups by specific time-phases. Box and whisker plots show the platelet parameters of the severe thrombocytopenia dengue group defined as platelet nadir $<50\times10^3$/µl during hospitalization and the non-severe thrombocytopenia dengue group observed in specific time-phases: febrile phase (day 1 to 3), critical phase (day 4 to 6), and recovery phase (day 7 to 10). Boxes show the median and interquartile values, whiskers represent the upper and lower adjacent values and dots indicate outside values. The comparison between each pair of dengue subgroups were performed using the Mann Whitney test. *: P<0.05, †: P<0.01, ‡: P<0.001. IPF%: Immature Platelet Fraction, IPF#: Immature Platelet Fraction Count.

Discussion

In this study we investigated the platelet parameters IPF% and IPF# in a dengue group at the point of admission and during the course of hospitalization. Our evaluation revealed unique platelet pattern parameters in the dengue group when compared with those of the confirmed and suspected CABI groups ~~of the same population~~. In brief, in a time course evaluation the IPF% of the dengue group was significantly higher than those of the confirmed and suspected CABI groups at admission and during the critical and recovery phases. When we considered only those with severe thrombocytopenia, the IPF% of the dengue group remained significantly higher than those of the confirmed (at admission, and in the critical and recovery phases) and suspected CABI groups (only in the recovery phase) even though their platelet levels were comparable in the same phases. Similarly, the IPF# of the confirmed CABI group was lower than that of the dengue group at admission and in the critical phase, with matching severity of thrombocytopenia.

It is assumed that an increased IPF% indicates a consumptive or destructive thrombocytopenic status and a normal or decreased IPF% suggests decreased platelet production in bone marrow. IPF# is assumed to reflect real-time platelet production [5,15,16]; however, one study claimed that IPF# changes should be approached with caution because fluctuations might be diminished by converting IPF% to absolute numbers in thrombocytopenia. [17] Clinical interpretations of IPF% and IPF# have been established based on observations mainly among hematologic disorders. For example, high IPF% was reported in ITP and TTP, which are caused by increased platelet consumption, while low to normal IPF% and low IPF# were documented in aplastic anemia and chemotherapy-induced thrombocytopenia, which are caused by decreased platelet production in bone marrow. [4,7,9] Low IPF# was observed in ITP with high IPF%, which is compatible with the fact that ITP is a multifactorial autoimmune disease characterized by both increased platelet destruction and/or reduced platelet production; and increased IPF# was observed in ITP when treated by a thrombopoietin receptor agonist which increases platelet production. [5,7] These observations support clinical

interpretations of IPF% and IPF#. Recently, IPF% and IPF# were further applied for infectious syndromes such as sepsis. [18-20]

Hypotheses proposed regarding the underlying mechanisms of dengue-induced thrombocytopenia are categorized mainly into two: decreased platelet production by bone marrow or increased peripheral platelet consumption or destruction. [21,22] In this study the IPF% of the dengue group was significantly higher than those of confirmed and suspected CABI groups in critical and/or recovery phases even after matching thrombocytopenia levels. Additionally, comparison between the severe thrombocytopenia and non-severe thrombocytopenia dengue groups showed a significantly higher IPF% of the severe thrombocytopenia dengue group versus those of other groups in the critical and recovery phases. Considering that IPF% reflects the balance of two potential mechanisms - increased platelet consumption or destruction with high IPF% and decreased platelet production with normal or low IPF% - these results might suggest the predominance of increased peripheral platelet consumption or destruction over decreased platelet production by bone marrow as an underlying mechanism of dengue-induced thrombocytopenia in the critical and recovery phases.

Moreover, decreased platelet production by bone marrow might potentially co-exist with peripheral platelet consumption or destruction at the peak of dengue-induced thrombocytopenia, because the comparison between the severe thrombocytopenia dengue and the non-severe thrombocytopenia dengue groups showed a significantly lower IPF# of the severe thrombocytopenia group versus those of other groups in the critical phase. Because the IPF# of the confirmed CABI group was significantly lower than that of dengue in the critical phase after matching the thrombocytopenia levels, the suppression of platelet production by bone marrow seems to be more predominant in CABI-induced than in dengue-induced thrombocytopenia.

Drawing from their use in hematologic disorders, IPF% and IPF# offer insight into dengue-induced thrombocytopenia as well as provide information for the clinical management of dengue patients. Our results show differences of IPF% and IPF# between dengue and the CABI groups and between severe and non-severe thrombocytopenia dengue groups in particular phases. IPF parameters are also useful to predict the recovery of platelets [23]; therefore, IPF% and IPF# are potentially valuable

parameters for the differential diagnosis of dengue from CABI, prediction of the severity of dengue-induced thrombocytopenia, and anticipation of platelet recovery.

This study has some limitations. Sequential day-by-day test results were not available for all patients, and this may introduce overestimation when severe patients were tested more frequently. There may be unreliability in patient reported onset day of symptoms. Detailed investigations were not available at the study site to determine possible primary or secondary dengue infections and dengue serotypes. We could not check serum thrombopoietin, the main factor for regulating thrombopoiesis. This was a single-center study and further data accumulation is required in various countries and clinical settings to validate our findings.

This study demonstrated the distinctive characteristics and time-course trends of IPF% and IPF# in a dengue group, which were ~~conspicuous when~~ compared with those of confirmed and suspected CABI groups. IPF% and IPF# are potentially valuable parameters in dengue and further investigation is required for the optimal ~~usage~~ in clinical practice.

Acknowledgements

We thank all the patients who participated in this study. We are grateful to the Director and staff of San Lazaro Hospital for their support in the conduct of this study. We thank Christopher M. Parry for commenting on previous drafts. We also wish to thank SLH-Nagasaki research staffs.

References

1. Simmons CP, Farrar JJ, Nguyen v V, Wills B. Dengue. N Engl J Med. 2012; 366: 1423–1432. 10.1056/NEJMra1110265. PMID: 22494122.
2. Dengue: Guidelines for Diagnosis, Treatment, Prevention and Control: New Edition. Geneva: 2009.
3. de Azeredo EL, Monteiro RQ, de-Oliveira Pinto LM. Thrombocytopenia in Dengue: Interrelationship between Virus and the Imbalance between Coagulation and Fibrinolysis and Inflammatory Mediators. Mediators Inflamm. 2015; 2015: 313842. 10.1155/2015/313842. PMID: 25999666.
4. Briggs C, Kunka S, Hart D, Oguni S, Machin SJ. Assessment of an immature platelet fraction (IPF) in peripheral thrombocytopenia. Br J Haematol. 2004; 126: 93–99. 10.1111/j.1365-2141.2004.04987.x. PMID: 15198738.
5. Barsam SJ, Psaila B, Forestier M, Page LK, Sloane PA, Geyer JT, et al. Platelet production and platelet destruction: assessing mechanisms of treatment effect in immune thrombocytopenia. Blood. 2011; 117: 5723–5732. 10.1182/blood-2010-11-321398. PMID: 21389318.
6. Kickler TS, Oguni S, Borowitz MJ. A clinical evaluation of high fluorescent platelet fraction percentage in thrombocytopenia. Am J Clin Pathol. 2006; 125: 282–287. 10.1309/50H8-JYHN-9JWC-KAM7. PMID: 16393688.
7. Abe Y, Wada H, Tomatsu H, Sakaguchi A, Nishioka J, Yabu Y, et al. A simple technique to determine thrombopoiesis level using immature platelet fraction (IPF). Thromb Res. 2006; 118: 463–469. 10.1016/j.thromres.2005.09.007. PMID: 16253312.
8. Jung H, Jeon HK, Kim HJ, Kim SH. Immature platelet fraction: establishment of a reference interval and diagnostic measure for thrombocytopenia. Korean J Lab Med. 2010; 30: 451–459. 10.3343/kjlm.2010.30.5.451. PMID: 20890075.
9. Sakuragi M, Hayashi S, Maruyama M, Kabutomori O, Kiyokawa T, Nagamine K, et al. Clinical significance of IPF% or RP% measurement in distinguishing primary immune thrombocytopenia from aplastic thrombocytopenic disorders. Int J Hematol. 2015; 101: 369–375. 10.1007/s12185-015-1741-0. PMID: 25618218.

10. Kitashoji E, Koizumi N, Lacuesta TL, Usuda D, Ribo MR, Tria ES, et al. Diagnostic Accuracy of Recombinant Immunoglobulin-like Protein A-Based IgM ELISA for the Early Diagnosis of Leptospirosis in the Philippines. PLoS Negl Trop Dis. 2015; 9: e0003879. 10.1371/journal.pntd.0003879. PMID: 26110604.
11. Lanciotti RS, Calisher CH, Gubler DJ, Chang GJ, Vorndam AV. Rapid detection and typing of dengue viruses from clinical samples by using reverse transcriptase-polymerase chain reaction. J Clin Microbiol. 1992; 30: 545-551. PMID: 1372617.
12. Nakao H, Popovic T. Development of a direct PCR assay for detection of the diphtheria toxin gene. J Clin Microbiol. 1997; 35: 1651-1655. PMID: 9196167.
13. Corless CE, Guiver M, Borrow R, Edwards-Jones V, Fox AJ, Kaczmarski EB. Simultaneous detection of Neisseria meningitidis, Haemophilus influenzae, and Streptococcus pneumoniae in suspected cases of meningitis and septicemia using real-time PCR. J Clin Microbiol. 2001; 39: 1553-1558. 10.1128/JCM.39.4.1553-1558.2001. PMID: 11283086.
14. Koizumi N, Nakajima C, Harunari T, Tanikawa T, Tokiwa T, Uchimura E, et al. A new loop-mediated isothermal amplification method for rapid, simple, and sensitive detection of Leptospira spp. in urine. J Clin Microbiol. 2012; 50: 2072-2074. 10.1128/JCM.00481-12. PMID: 22422858.
15. Koyama K, Katayama S, Muronoi T, Tonai K, Goto Y, Koinuma T, et al. Time course of immature platelet count and its relation to thrombocytopenia and mortality in patients with sepsis. PLoS One. 2018; 13: e0192064. 10.1371/journal.pone.0192064. PMID: 29381746.
16. Bat T, Leitman SF, Calvo KR, Chauvet D, Dunbar CE. Measurement of the absolute immature platelet number reflects marrow production and is not impacted by platelet transfusion. Transfusion. 2013; 53: 1201-1204. 10.1111/j.1537-2995.2012.03918.x. PMID: 23043309.
17. Takami A, Shibayama M, Orito M, Omote M, Okumura H, Yamashita T, et al. Immature platelet fraction for prediction of platelet engraftment after allogeneic stem cell transplantation. Bone Marrow Transplant. 2007; 39: 501-507. 10.1038/sj.bmt.1705623. PMID: 17334382.

18. De Blasi RA, Cardelli P, Costante A, Sandri M, Mercieri M, Arcioni R. Immature platelet fraction in predicting sepsis in critically ill patients. Intensive Care Med. 2013; 39: 636–643. 10.1007/s00134-012-2725-7. PMID: 23093245.

19. Enz Hubert RM, Rodrigues MV, Andreguetto BD, Santos TM, de Fatima Pereira Gilberti M, de Castro V, et al. Association of the immature platelet fraction with sepsis diagnosis and severity. Sci Rep. 2015; 5: 8019. 10.1038/srep08019. PMID: 25620275.

20. Muronoi T, Koyama K, Nunomiya S, Lefor AK, Wada M, Koinuma T, et al. Immature platelet fraction predicts coagulopathy-related platelet consumption and mortality in patients with sepsis. Thromb Res. 2016; 144: 169–175. 10.1016/j.thromres.2016.06.002. PMID: 27380496.

21. Hottz E TN, Zimmerman G, Weyrich A, Bozza F. Platelets in dengue infection. Drug Discov Today Dis Mech. 2011; 8 1-2(pg. e33-e38).

22. Hottz ED, Bozza FA, Bozza PT. Platelets in Immune Response to Virus and Immunopathology of Viral Infections. Front Med (Lausanne). 2018; 5: 121. 10.3389/fmed.2018.00121. PMID: 29761104.

23. Dadu T, Sehgal K, Joshi M, Khodaiji S. Evaluation of the immature platelet fraction as an indicator of platelet recovery in dengue patients. Int J Lab Hematol. 2014; 36: 499–504. 10.1111/ijlh.12177. PMID: 25356498.

Supporting information

S1 Fig. Comparison of platelet, IPF%, and IPF# among dengue and each confirmed CABI at admission. The lines show the median with interquartile ranges. Comparisons of each group were performed using the Kruskal-Wallis test followed by the Dunn’s post hoc test with Holm adjustment. *: P<0.05, †: P<0.01, ‡: P<0.001. IPF%: Immature Platelet Fraction, IPF#: Immature Platelet Fraction Count.

S2 Fig. Time course trends of platelet parameters in each group from the first to tenth days of illness (Line plot). Data are expressed as mean with a 95% confidence interval shown by the error bars. The total number of evaluations included on each day is indicated on the X axis by each group. The mean of each platelet parameter by days of illness were calculated only from the results of participants who underwent a blood test on each day. CABI: community acquired bacterial infection, IPF%: Immature Platelet Fraction, IPF#: Immature Platelet Fraction Count.

S3 Fig. Time course of platelet parameters in the dengue group by age groups. Box and whisker plots show the time course trends of platelet parameters in the dengue group by age from the first to tenth days of illness. Boxes show the median and

interquartile values, whiskers represent upper and lower adjacent values and dots indicate outside values. The total number of evaluations included on each day is indicated on the X axis by each group. IPF%: Immature Platelet Fraction, IPF#: Immature Platelet Fraction Count.

S4 Fig. Comparison of platelet parameters between the severe and non-severe dengue groups by specific time-phases. Box and whisker plots show the platelet parameters of severe and non-severe dengue groups observed in specific time-phases; febrile phase (day 1 to 3), critical phase (day 4 to 6) and recovery phase (day 7 to 10). Severe dengue was clinically defined in the “Disease definition” section. Boxes show the median and interquartile values, whiskers represent the upper and lower adjacent values and dots indicate outside values. Comparisons between each pair of dengue subgroups were performed using the Mann Whitney test. IPF%: Immature Platelet Fraction, IPF#: Immature Platelet Fraction Count.

S1 Table. Comparison of the results from real-time PCR (*rrs* real-time PCR) and *flaB*-nested PCR. A total number of 50 negative samples and 43 positive samples for *flaB*-nested PCR were tested in duplicate by *rrs* real-time PCR. The 50 negative

samples for *flaB*-nested PCR were also negative for the microscopic agglutination test using paired sera. This *rrs* real-time PCR did not detect bacterial species other than *Leptospira* spp. described in published study. [14] *rrs*: 16S ribosomal RNA gene.

Fig1

Click here to access/download;Figure;Fig1.tif

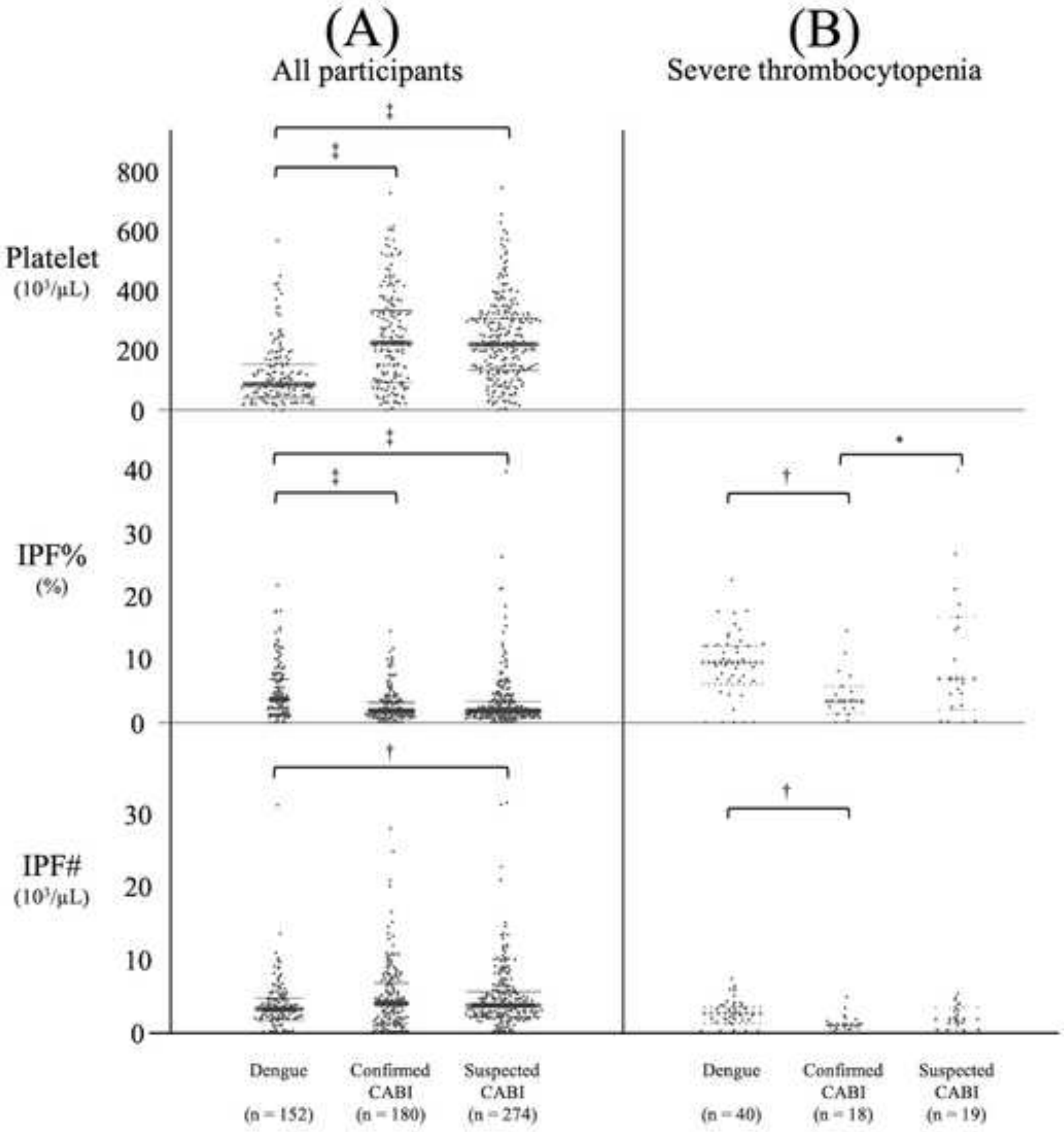

Fig2 Click here to access/download;Figure;Fig2.tif

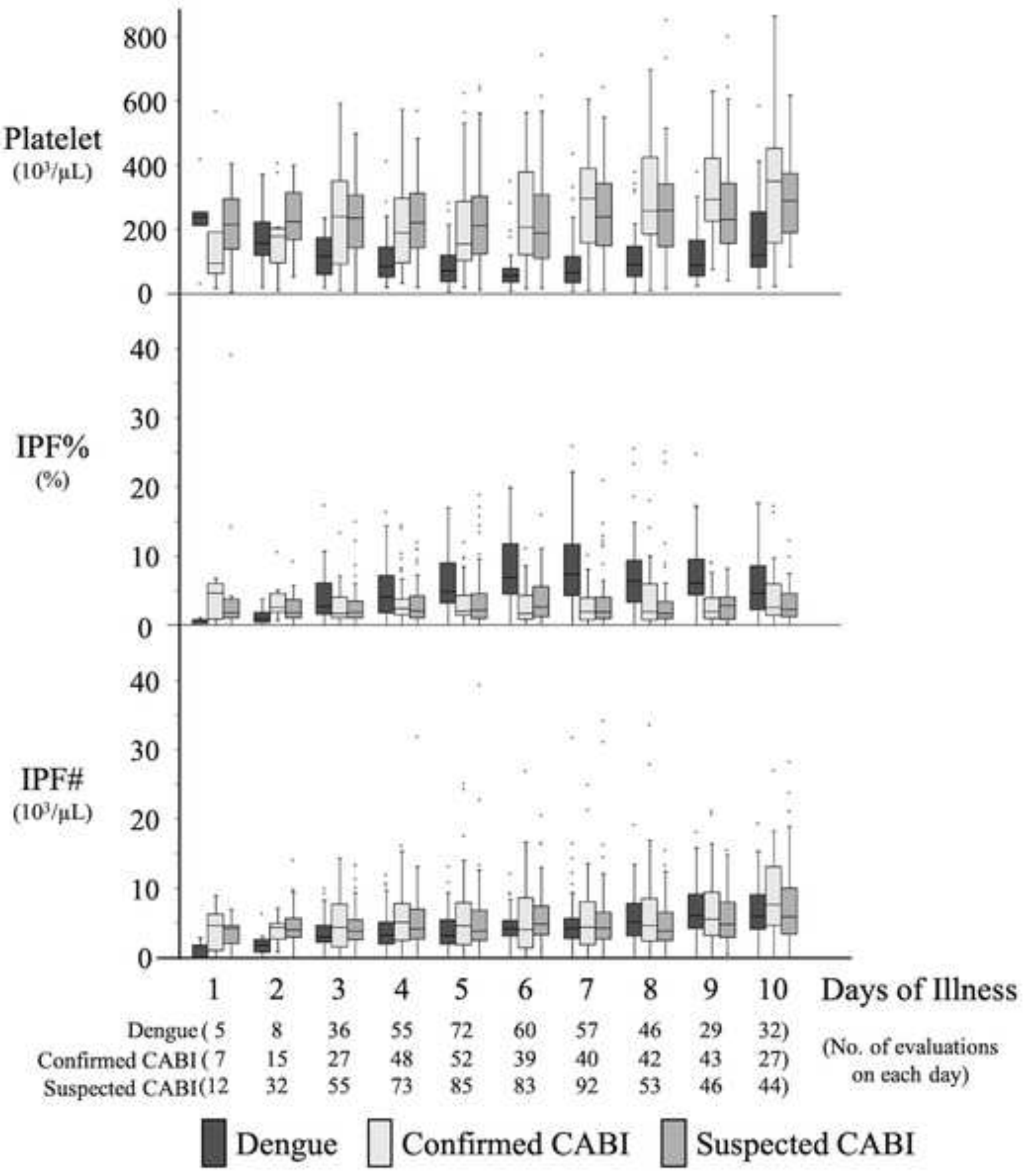

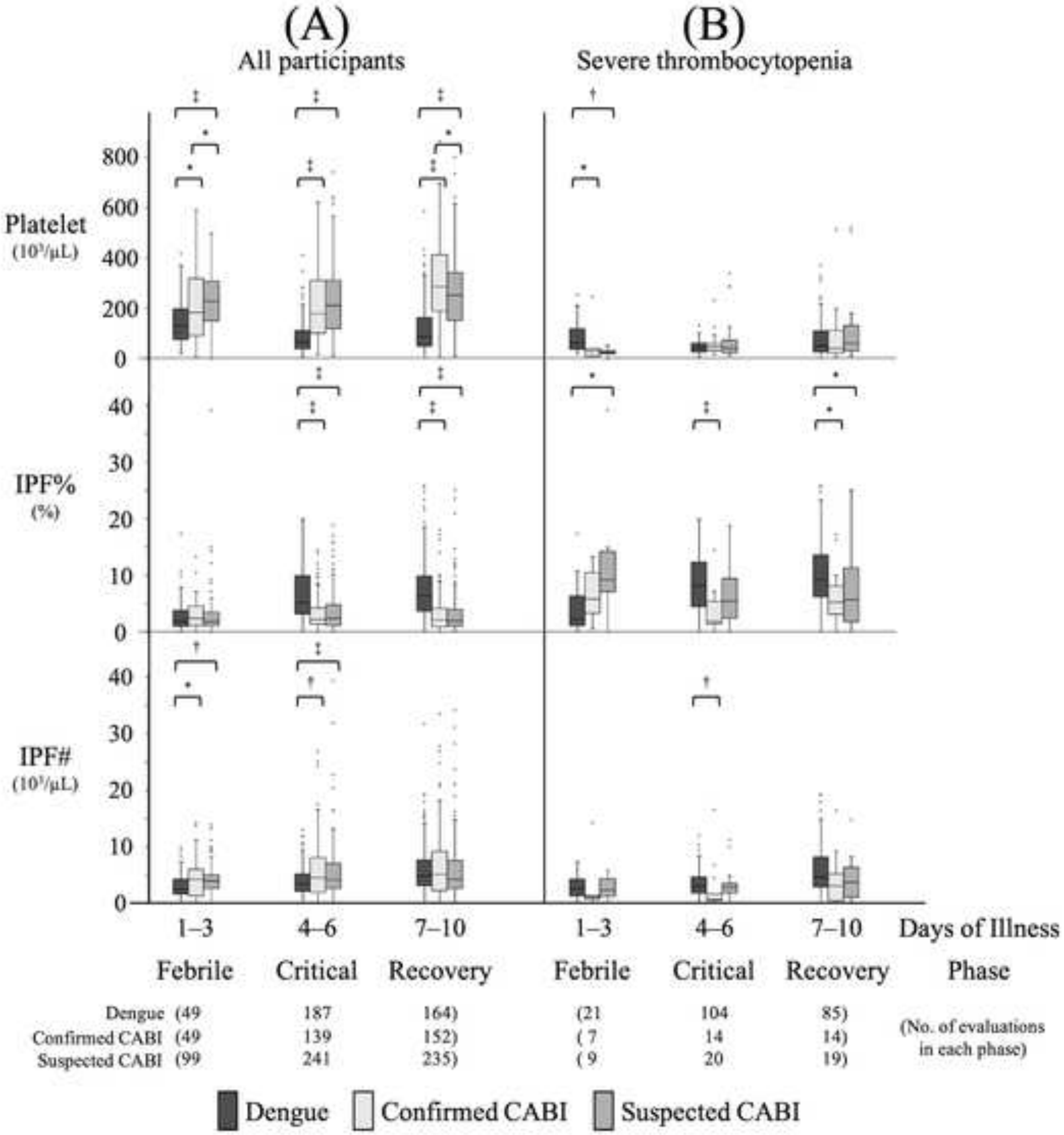
(A)
All participants
(B)
Severe thrombocytopenia
Platelet
(10³/μL)
IPF%
(%)
IPF#
(10³/μL)
1–3
4–6
7–10
Days of Illness
Febrile
Critical
Recovery
Phase
Dengue (49 187 164) (21 104 85)
Confirmed CABI (49 139 152) (7 14 14)
Suspected CABI (99 241 235) (9 20 19)
(No. of evaluations in each phase)
Dengue
Confirmed CABI
Suspected CABI

 Click here to access/download;Figure;Fig4.tif

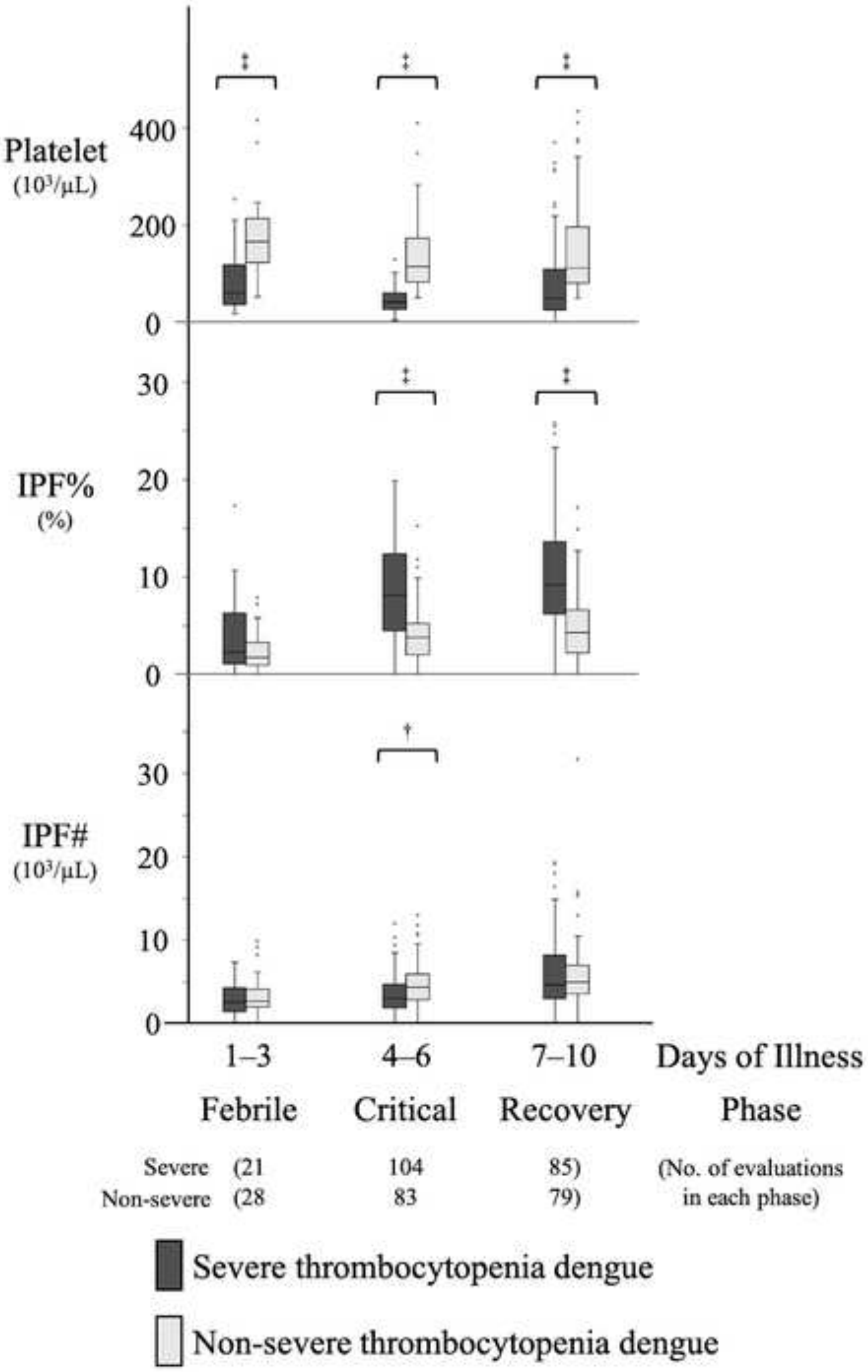

S1_Fig

Click here to access/download
Supporting Information
S1_Fig.tif

Click here to access/download
Supporting Information
S1_Table.docx

S2_Fig

Click here to access/download
Supporting Information
S2_Fig.tif

S3_Fig

Click here to access/download
Supporting Information
S3_Fig.tif

S4_Fig

Click here to access/download
Supporting Information
S4_Fig.tif
