# Supplementary material for: Correction: Unique characteristics of new complete blood count parameters, the Immature Platelet Fraction and the Immature Platelet Fraction Count, in dengue patients
Source: PLoS One. 2024 May 2;19(5):e0303463. doi: 10.1371/journal.pone.0303463 (PMC11065236; doi:10.1371/journal.pone.0303463)
Supplement: S1 Appendix — (PDF) [file pone.0303463.s001.pdf]

S1 Appendix. Diagnostic criteria of CABIs.

Bacteremia was diagnosed by positive blood culture. Diphtheria was diagnosed when diphtheria culture and/or tox-gene PCR from nasopharyngeal or oropharyngeal sample were positive. Meningococcal disease was diagnosed when meningococcus culture and/or PCR from blood or skin lesion samples were positive. Leptospirosis was diagnosed when one of the following were positive: culture with Korthof's medium, Patoc-IgM enzyme linked immunosorbent assay (ELISA) (positivity was defined as described [13]), or real-time PCR assay (see the details described in S2 appendix). X-ray confirmed pneumonia was diagnosed when patients had acute onset of respiratory symptoms and radiographic shadowing in lung fields without other suspected infection focus or positive blood culture result. Skin infection was diagnosed when patients had acute onset of symptoms compatible with skin infection without other suspected infection focus or positive blood culture result.
