# Supplementary material for: Correction: Unique characteristics of new complete blood count parameters, the Immature Platelet Fraction and the Immature Platelet Fraction Count, in dengue patients
Source: PLoS One. 2024 May 2;19(5):e0303463. doi: 10.1371/journal.pone.0303463 (PMC11065236; doi:10.1371/journal.pone.0303463)
Supplement: S2 Appendix — (PDF) [file pone.0303463.s002.pdf]

S2 Appendix. Laboratory procedure for diagnoses of CABIs.

The blood culture results were detected using a BacT/ALERT® automated system (BioMerieux) and isolates were identified by Matrix Assisted Laser Desorption/Ionization Time of Flight Mass Spectrometry (MALDI-TOF MS) or standard microbiological methods. Primer pairs and probes that target leptospiral 16S ribosomal RNA gene (*rrs*) were newly designed for a real-time PCR assay (F3C, 5'-TCATTGGGCGTAAAGGGTG-3'; B3C, 5'-TCAGTTTTAGGCCAGCAAGTC-3'; Probe, 56-FAM/ AGAGGCAAG/ZEN/TGGAATTCCAGGTG/BHQ), and the performance compared with *flaB* nested PCR as shown in S1 Table. The details of PCR and ELISA method used in this study were as published. [13-17]
